# Pharmacokinetic and pharmacodynamic study of doxorubicin in children with cancer: results of a “European Pediatric Oncology Off-patents Medicines Consortium” trial

**DOI:** 10.1007/s00280-016-3174-8

**Published:** 2016-10-21

**Authors:** Miriam Krischke, Georg Hempel, Swantje Völler, Nicolas André, Maurizio D’Incalci, Gianni Bisogno, Wolfgang Köpcke, Matthias Borowski, Ralf Herold, Alan V. Boddy, Joachim Boos

**Affiliations:** 1 Pediatric Hematology and Oncology, University Hospital Münster, Albert-Schweitzer-Campus 1, Building A1, 48149 Münster, Germany; 2 Zentrum für Klinische Studien (ZKS), University Hospital Münster, Von-Esmarch-Straße 62, 48149 Münster, Germany; 3 Department of Pharmaceutical and Medical Chemistry, Clinical Pharmacy, Westfälische Wilhelms-Universität Münster, Corrensstraße 48, 48149 Münster, Germany; 4 INSERM UMR 911, Centre de Recherche en Oncologie biologique et en Oncopharmacologie, Aix-Marseille University, Marseille, France; 5 Hematology and Pediatric Oncology Department, La Timone University Hospital of Marseille, Marseille, France; 6 Department of Oncology, IRCCS - Istituto di Ricerche Farmacologiche Mario Negri, Via La Masa, 19, 20156 Milan, Italy; 7 Dipartimento di Pediatria, Clinica di Oncoematologia, via Giustiniani, 3, 35100 Padua, Italy; 8 Institute of Biostatistics and Clinical Research, Westfälische Wilhelms-Universität Münster, Schmeddingstraße 56, 48149 Münster, Germany; 9 European Medicines Agency (EMA), 30 Churchill Place, Canary Wharf, London, E14 5EU UK; 10 Northern Institute for Cancer Research, Newcastle University, Paul O’Gorman Building, Medical School, Framlington Place, Newcastle upon Tyne, NE2 4HH UK; 11 Faculty of Pharmacy, University of Sydney, Sydney, Australia

**Keywords:** Doxorubicin, Cancer, Children, Pharmacokinetic, Cardiotoxicity

## Abstract

**Purpose:**

Doxorubicin is a key component in many pediatric oncology treatment regimens; still pharmacology data on which current dosing regimens are based are very limited.

**Methods:**

We conducted a multinational pharmacokinetic study investigating age dependency of doxorubicin metabolism and elimination in children with cancer. One hundred and one patients treated with doxorubicin according to a cancer-specific national or European therapeutic trial were recruited. Doses of doxorubicin ranged from 10.4 to 57.7 mg/m^2^. Blood samples for measurement of doxorubicin and its metabolite doxorubicinol were collected after two administrations, with five samples collected in children <3 years and eight in children ≥3 years. A population pharmacokinetic approach was used for analysis, including pharmacogenetic covariates. Natriuretic peptides and cardiac troponins were measured to evaluate their role as early indicators of cardiotoxicity.

**Results:**

Age dependence of doxorubicin clearance was demonstrated, with children less than 3 years having a statistically significant lower clearance (21.1 ± 5.8 l/h/m^2^) than older children (26.6 ± 6.7 l/h/m^2^) (*p* = 0.0004) after correcting for body surface area. No effect of the investigated genetic polymorphisms on the pharmacokinetics could be observed. Although natriuretic peptides were transiently elevated after each doxorubicin administration and troponin levels increased with increasing doxorubicin exposure, only limited correlation could be observed between their blood levels and doxorubicin pharmacokinetics.

**Conclusion:**

In the European framework of funding and regulatory support, an add-on study to existing therapeutic trials was developed. The pediatric need concerning missing PK data could be addressed with limited burden for the patients. Empirically used dose adaptations for infants were generally found to be justified based on our PK analyses.

**Electronic supplementary material:**

The online version of this article (doi:10.1007/s00280-016-3174-8) contains supplementary material, which is available to authorized users.

## Introduction

Doxorubicin is included in a wide variety of cancer treatment protocols for children and adults. As with other anticancer agents, doxorubicin has a narrow therapeutic window and serious side effects, including myelosuppression, mucositis and cardiotoxicity. Late-onset cardiotoxicity presents a major problem of doxorubicin therapy since it might lead to congestive heart failure years after end of therapy sometimes without prior clinical symptoms.

Late subclinical effects, i.e., abnormal left ventricular structure and function, might affect more than 50 % of childhood cancer survivors, whereas clinical events might occur to 1–10 % [1]. Known risk factors include cumulative dosage, younger age, use of concomitant cardiotoxic therapies, female sex and higher dose intensity [2]. Several genetic markers such as SLC28A3 and UGT1A6 are also discussed [3, 4].

Standard echocardiographic monitoring is not sensitive enough to detect early subclinical deteriorations of the heart, and the positive predictive value is not sufficient for late heart failure. Therefore, more sensitive markers are necessary. Potential candidates are the cardiac isoforms of troponins, cardiac troponin T (cTnT) and I (cTnI), that are specific and sensitive biomarkers of myocardial cell injury [5–7], as well as the natriuretic peptides ANP (A-type natriuretic peptide) and BNP (B-type natriuretic peptide). The latter as well as their precursors NT-proANP (N-terminal-proANP) and NT-proBNP (N-terminal-pro BNP) are peptide hormones that are secreted by the atria or ventricle after increased pressure on the heart wall and myocyte stretch [8, 9].

Despite its frequent use, the pharmacokinetics of doxorubicin have never been studied systematically in children. There are only few studies in the literature, and those have included only low numbers of patients and used limited sampling schedules.

The therapeutic window for doxorubicin dosing is narrow, and a better understanding of doxorubicin pharmacokinetics in children is thus crucial specifically for the very young. This deficit in information for the safe use of doxorubicin encouraged the European Medicines Agency (EMA) to place doxorubicin on the “Priority List” (Doc. Ref. EMEA/197972/2007, London, June 2007) for medications with a high priority for further research on pediatric use of the medicinal product. This list was a basis for application and subsequent funding within the European Union 7th Framework call “HEALTH-2007-4.2-1: Adapting off-patent medicines to the specific needs of pediatric populations.” Within that call, the European Pediatric Oncology Off-patent Medicines Consortium (EPOC) applied and obtained funding. The consortium organized a European multinational and multicenter trial called the “Doxo”-study.

The *primary objective* was to evaluate age dependency in pharmacokinetics of doxorubicin in children and adolescents. As *secondary objectives*, genetic polymorphisms that could influence doxorubicin clearance were investigated as well as the potential role of natriuretic peptides and troponin as indicators for subclinical cardiotoxicity.

## Patients and methods

### Study population

Children with solid tumors or leukemia were enrolled between April 2010 and October 2012 in 20 hospitals from four European countries (Germany, France, Italy and UK). Eligible patients were younger than 18 years and were planned to receive two or more cycles of doxorubicin for treatment of their disease. To be eligible, children three years and older had to be enrolled in a national or European protocol for treatment of Wilms tumor, neuroblastoma, soft tissue sarcoma, Ewing sarcoma or acute lymphoblastic leukemia. Children younger than three years had to be enrolled or listed in any national or European study protocol for any pediatric malignancy. Additional blood withdrawal had to be acceptable for the patient, and assent was obtained from patients deemed to be able to assent. Written informed consent was obtained from their parents or legal guardians.

### Study organization

Sponsorship for the EPOC-MS-001-Doxo-Trial (EudraCT-Nr: 2009-011454-17, ClinicalTrials.gov Identifier: NCT01095926) was provided by the University Hospital Münster, Germany, and national study managers (NSM) set up the clinical network and dealt with regulatory and ethical issues in their respective countries. An independent Data Monitoring Committee reviewed patient safety data and overall data quality. The study was run in accordance with the Declaration of Helsinki, in compliance with the International Conference on Harmonization Good Clinical Practice (GCP) Guidelines and applicable local regulatory requirements and laws. The study was approved by all nationally and locally responsible ethics committees.

### Study endpoints

The primary endpoint was to determine whether there is a difference in doxorubicin clearance between children <3 years and children 3 to <18 years. Additional analyses were performed to assess the interindividual, intraindividual and residual variability of doxorubicin pharmacokinetic (PK) parameters in children and to assess the relationship between PK parameters and patient characteristics, including genetic polymorphisms that may influence doxorubicin clearance (CL). Furthermore, kinetics of natriuretic peptides and troponins were measured to explore their potential role as indicators for subclinical cardiotoxicity.

### Doxorubicin administration

Patients received doxorubicin intravenously according to the treatment protocol for their tumor entity. Infusion durations ranged from 30 min to 48 h and mirrored usual clinical practice (see Online Resource 2 for more information on doxorubicin dosage and infusion times used in the various treatment regimens). To harmonize practical issues concerning doxorubicin administration, a precise working procedure was provided to the participating centers. Herein, the use of a syringe-driven infusion system or at least a system with a line volume from infusion bag to patient being less than 10 % of the doxorubicin volume was mandated.

### PK, biomarker and pharmacogenetic assessments

Blood for analysis of doxorubicin and doxorubicinol concentrations was obtained during and up to 24 h after doxorubicin administration during two cycles chosen by the investigator. A pharmacokinetic dataset of five samples [3 + 2 (first + second sampling period)] was collected in children <3 years and eight samples (5 + 3) in older children. The first sample of each cycle was taken during doxorubicin infusion and had to be taken by capillary or peripheral blood sampling, whereas all other samples were taken after the end of doxorubicin administration and could thus be taken from the central venous catheter (CVC) which was also used for doxorubicin administration. Clear guidelines for CVC cleaning and blood sampling had to be followed. Blood was collected into EDTA (ethylenediaminetetraacetate) tubes, put immediately on ice and centrifuged within 15 min at 1600*g* for 5 min at 4 °C. Plasma had to be kept at −20 °C or lower until analysis. Samples were sent from the clinical centers to the NSM for quality checks and from there passed on to the laboratory in Münster for analysis.

Doxorubicin and doxorubicinol concentrations were measured using a validated HPLC (high-performance liquid chromatography) method (as described in detail before [10]) and were analyzed by population pharmacokinetic methods using nonlinear mixed-effects modeling (NONMEM) [11].

**Fig. 1:**
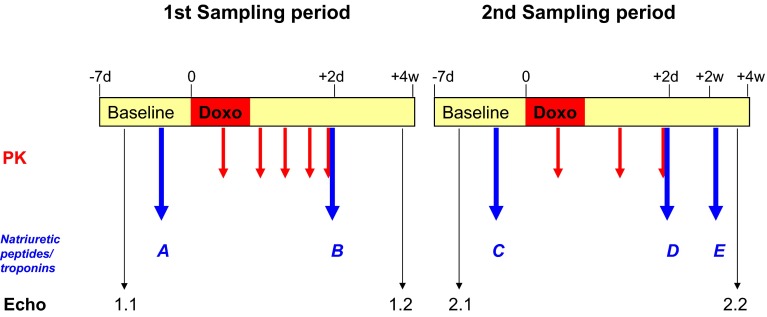
Study design (sampling scheme for patients >3 years). Simplified overview of the main trial procedures, including pharmacokinetic and natriuretic peptides/troponin sampling times (termed *A*–*E*) as well as echocardiography schedules (termed 1.1–2.2). Sampling had to be done from two different chemotherapy blocks chosen by the investigator. Sampling schemes for patients <3 years were identical, with the exception that only 5 PK samples (3 + 2) were requested

Natriuretic peptides and cardiac troponins were measured in plasma using commercially available enzyme-linked immunosorbent assay kits (cTnT: troponin T hs, Roche Diagnostics GmbH, Mannheim, Germany/proANP (1–98): Biomedica Medizinprodukte, Wien/cTnI: TnI-Ultra, ADVIA Centaur, BNP: ADVIA Centaur and NT-proBNP: Immulite 2500, all three from Siemens Healthcare Diagnostics, Tarrytown, NY, USA). Samples were taken before doxorubicin administration and together with the last PK sample of each cycle. A fifth sample was taken 2–4 weeks after doxorubicin administration in the second sampling cycle (Fig. 1). Blood was collected into EDTA tubes and centrifuged at 1600*g* for 5 min at room temperature. Plasma had to be kept at −20 °C or lower until analysis.

Whole-blood (EDTA) samples for pharmacogenetic analysis were collected, frozen and shipped to the laboratory in Newcastle for analysis.

### Echocardiographic measurements

Left ventricular shortening fraction (LVSF) was determined by echocardiograms before and after the two investigated doxorubicin administrations. “After” was defined as before the next protocol block containing doxorubicin, but not later than 28 days after doxorubicin administration.

### Statistical analysis

The primary objective was to compare the distributions of the BSA-normalized clearance of the two age groups (group A: <3 years, group B: 3 to <18 years). The sample size calculation was based on the assumption that the relative clearance in both age groups follows a log-normal distribution. Since Frost et al. [12] found a between-subject variation in clearance/m^2^ (CL_BSA_) of 46 %, the standard deviation was assumed to be 0.46 in both groups. Given at least 20 patients in age group A and 80 patients in age group B, the calculated power to detect a difference of 39 % was 90 % for a two-sided unpaired *t* test with level of significance 5 %. During analysis, the empirical distributions of the logarithmized and non-logarithmized CL_BSA_ values suggested that the assumption of normality was more justifiable for the original, non-logarithmized CL_BSA_ values. We therefore performed the *t* test on the original CL_BSA_ data. Since the empirical variances of the CL_BSA_ values differed considerably between age group A and B, Welch’s *t* test for groups with unequal variances was applied.

The statistical analyses of the secondary objectives were carried out using statistical key figures like mean and standard deviation, empirical quantiles and Spearman’s rank correlation coefficient, as well as scatter and box-and-whisker plots.

Moreover, statistical tests were performed and are reported to be (statistically) *noticeable* if a *p* value is smaller than 5 %. The term *significant* is avoided since the *p* values resulted from explorative analyses and were not adjusted for multiple testing. All statistical analyses were conducted using the statistical software R [13] and validated by at least one further statistician.

## Results

### Trial conduction and patient characteristics

In total, 110 patients consented to participate in the trial with nine patients being excluded before any baseline data were recorded and plasma samples taken. From the 101 patients, 94 patients contributed to PK analysis and 98 to biomarker and pharmacogenetic analysis. An overview of the patient demographics and clinical characteristics is presented in Table 1. Although patients with abnormal liver function were eligible for the study, none of the recruited patients had a serum bilirubin level above 1.2 mg/100 ml, which would have required a reduction of doxorubicin dose.

**Table 1:** Demographics and clinical characteristics of recruited patients

	Total	<3 years	3 to <18 years
No. of patients	101	27	74
*Age (years)*			
Median	5.3	1.6	7.3
Range	0.2–17.7	0.2–2.9	3.0–17.7
*Height (cm)*			
Median	111.5	84	129
Range	52–194	52–97	89–194
*Weight (kg)*			
Median	19.2	11.1	26
Range	3.6–88.1	3.6–14.7	111.5–88.1
*Doxorubicin dose (mg/m* ^*2*^ *)*			
Median	28.7	29.1	28.6
Range	10.4–57.7	10.4–57.7	18.2–56.8
*Doxorubicin infusion duration (h)*			
Median	3.88	3.95	3.88
Range	0.25–24.02	0.25–24	0.28–24.02
*Gender*			
Male	50	11	39
Female	51	16	35
*Tumor type*			
Wilms tumor	12	3	9
Neuroblastoma	9	4	5
Ewing sarcoma	27	1	26
Soft tissue sarcoma	16	5	11
ALL	30	7	23
Other	7	7	0
*Country*			
France	24	6	18
Germany	29	6	23
Italy	27	10	17
UK	21	6	15

Seven patients dropped out after the first sampling period. Reasons were disease progression or relapse (*n* = 4), problems with blood sampling from central venous catheter (*n* = 1) or withdrawal of consent (*n* = 2) (see Online Resource 1).

### Doxorubicin pharmacokinetic analysis

Determination of the optimal PK sampling time points for the trial was based on a model derived from adult data [14]. An initial population pharmacokinetic model for doxorubicin and doxorubicinol in children was set up from datasets of three earlier studies (two adults and one child) using NONMEM^®^ 7.2. The model-building strategy for children >3 years has been described in detail before [11]. A pre-planned interim analysis was performed after 30 patients to check the chosen sampling time points and readdress the number of samples and patients. The analysis did not lead to any protocol changes or amendments.

At the end of the trial, the model from the interim analysis was further adjusted to deliver a final PK model for doxorubicin and doxorubicinol in children. The final doxorubicin model was a three-compartment model scaled on BSA with an additional power function of age on the CL of the parent compound. An additional compartment for doxorubicinol was added to the final doxorubicin model in order to describe the PK of the metabolite. No covariate was able to improve the doxorubicinol model. A more detailed description of the model-building process was recently published [15].

**Fig. 2:**
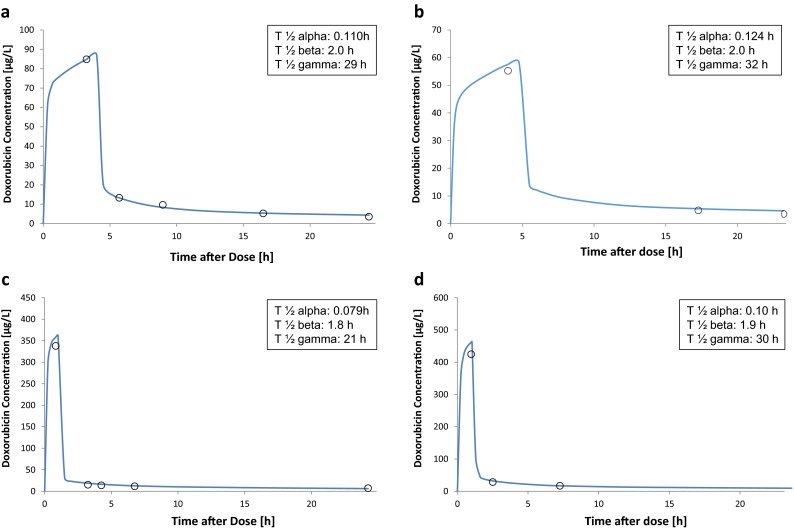
Plasma concentration time curves. Doxorubicin plasma concentration time curves estimated by the PopPK model of the first sampling period for four different patients are shown together with the corresponding doxorubicin plasma concentration half-lives. The *open circles* present the actual measured values. A and B were patients with soft tissue sarcoma and a scheduled infusion duration of 4 h. Patient A was 12.99 years old and patient B 0.66 years. The actual infusion time for patient B was 4.77 h. C and D were patients with ALL and a scheduled infusion duration of 1 h. Patient C was 12.89 years and patient D 2.09 years. The actual infusion time for D was 1.04 h

Doxorubicin plasma concentration time curves estimated by the PK model for four different patients are shown in Fig. 2. To show that the model was able to predict actual plasma concentrations for different infusion durations as well as for patients of different ages, we choose two different diseases (soft tissue sarcoma and ALL) with different infusion durations (4 and 1 h, respectively) and for each picked one patient below 3 years of age and one older than 12 years. The open circles represent the actual measured values. For each patient, the corresponding distribution, elimination and terminal phase elimination half-lives are listed. Mean half-lives for all patients were: *T*_½_ *α*: 0.101 h, *T*_½_ *β*: 2.0 h, *T*_½_ *γ*: 31 h.

**Fig. 3:**
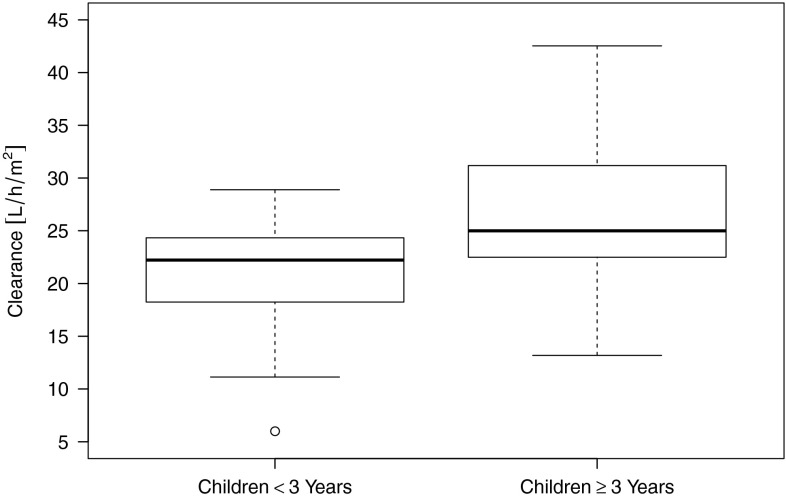
Age dependency of doxorubicin clearance. Distribution of clearance (CL) (normalized to BSA) for the two investigated age groups (A: <3 years and B: 3 to <18 years). Data are summarized as *box*-and-*whisker plots*. In each plot, the *central box* represents values from the lower to upper quartile (25–75 percentiles) and the *middle line* the median. The end of the *upper whisker* corresponds to the largest observation smaller than the 75 % quantile + 2*IQR. Likewise, the end of the *lower whisker* corresponds to the smallest observation larger than the 25 % quantile − 2*IQR. Observations that are beyond the whiskers can be regarded as outliers

The mean value for CL_BSA_ of both sampling periods was calculated for each patient using the NONMEM output tables. Comparison of CL_BSA_ between the two age groups (group A: <3 years, group B: 3 to <18 years) using the two-sided unpaired *t* test delivered a *p* value of 0.0004 indicating a significant difference. The mean CL_BSA_ is 26.6 L/h/m^2^ in age group A and 21.1 l/h/m^2^ in age group B, indicating that the CL of children <3 years is lower than the relative clearance of children between 3 and 18 years (see Fig. 3).

Two further tests were performed to confirm this result by means of a sensitivity analysis. Welch’s *t* test on the logarithmized CL_BSA_ data showed a *p* value of around 0.0039, and the *p* value of the nonparametric Mann–Whitney *U* test was approximately 0.0026. That is, both tests confirm the result of a significant difference between age groups A and B as to the CL_BSA_ distribution.

### Influence of various genetic polymorphisms on doxorubicin clearance

The influence of several known genetic polymorphisms occurring in eight genes encoding for proteins involved in doxorubicin metabolism or transport (ABCB1, ABCC1, CBR1, CBR3, NQ01, SLC22A16, SLC28A3 and UGT2BT) was analyzed. The percentage of homozygous individuals ranged from 0 to 26, 5 % [mean: 10.1 %], the one of heterozygous individuals from 10.2 to 59.2 % [mean: 34.9 %]. Although we detected quite a high number of polymorphisms and although increasing evidence exists that doxorubicin-induced cardiotoxicity can be predicted by pharmacogenomics analysis [3, 4], no influence of any of the investigated gene polymorphisms on the doxorubicin pharmacokinetics could be observed in the population PK model. An overview of the investigated polymorphisms along with the numbers of individuals being of wild-type, heterozygous or homozygous genotype is found in Online Resource 3.

### Natriuretic peptides and cardiac troponins as markers for doxorubicin-induced subclinical cardiotoxicity

Pre- and post-dose levels of both sampling periods (SP) were compared for two troponins (cTnT and cTnI) and three natriuretic peptides (BNP, NT-proBNP and NT-proANP) to determine whether the biomarker blood concentrations showed an increase after administration of doxorubicin. Post-dose levels (samples B and D) were drawn up to 2 days after end of doxorubicin administration with more than 50 % of the samples drawn between 22 and 25 h after end of administration (time after dose, mean sample (B): 23.8 h; mean sample (D): 21.6 h). A clear correlation was observed between the three natriuretic peptides, with the highest correlation between NT-proBNP and BNP and a slightly lower correlation between NT-proANP and the two BNP variants (Online Resource 4). A strong correlation could also be detected between the two examined troponins, whereas only medium to low correlations exist between the troponins and the natriuretic peptides.

**Fig. 4:**
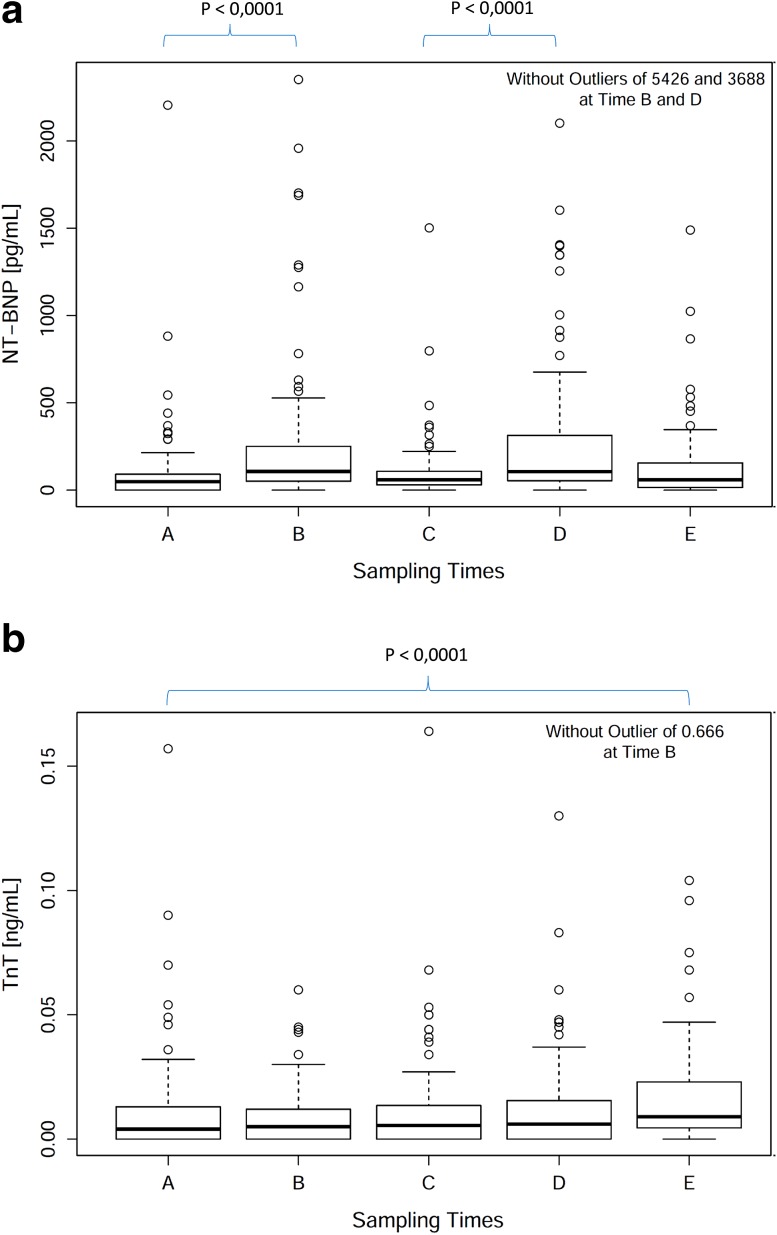
Biomarker concentrations of NT-proBNP and cTnT before and after doxorubicin administration. NT-proBNP (**a** exemplary for the three natriuretic peptides) and cTnT (**b** exemplary for the two cardiac troponins) concentrations in plasma samples before (Samples *A* and *C*) and at different times after doxorubicin administration (samples *B*, *D*, *E*). Data are summarized as *box*-and-*whisker plots* as explained in Fig. 3

Blood concentrations for all three natriuretic peptides increased noticeably compared to pre-dose levels (*p* < 0.001 for both the first and second SP for all three natriuretic peptides, Wilcoxon’s signed rank test) (Fig. 4a, NT-proBNP, exemplary for the three natriuretic peptides). In contrast, cTnT or cTnI was not noticeably elevated immediately after doxorubicin administration (cTnT: first SP *p* = 0.764, second SP *p* = 0.576; cTnI: first SP *p* = 0.951, second SP *p* = 0.035), but 2–4 weeks after the second sampling cycle (cTnT: *p* < 0.001, cTnI < 0.01), showing a delayed increase in cardiac troponin blood concentrations (Fig. 4b, cTnT, exemplary for both cardiac troponins).

Cardiac troponin blood concentrations correlated with administered cumulative doxorubicin dose normalized to BSA (cTnT: Spearman’s correlation: 0.409, *p* value <0.001; cTnI: Spearman’s correlation: 0.452, *p* value <0.001). A minor increase in blood concentration between sample A and E was also observed for NT-proBNP (*p* = 0.026), but not for the other two measured natriuretic peptides (BNP: *p* = 0.449; NT-proANP: *p* = 0.716). Furthermore, a marginal correlation to cumulative dose was observed for BNP (Spearman’s correlation: 0.371, *p* value <0.001), NT-proBNP (Spearman’s correlation: 0.242, *p* value: 0.023) and NT-proANP (Spearman’s correlation: 0.256, *p* value = 0.016).

We furthermore assessed the correlation between the pre–post-differences in natriuretic peptide concentrations (i.e., before and after doxorubicin administration for each sampling period) and the individual doxorubicin dose intensity in each cycle (Table 2) represented by the AUC (area under the curve) and *C*_max_. No correlation could be detected for Doxo AUC, but small negative correlations were detected between the concentration differences of all natriuretic peptides and *C*_max_. Since the correlation coefficients are small, the tests were not adjusted for multiple testing and a lot of confounder might interfere with this observation, these differences are difficult to explain and might also be arisen by chance.

**Table 2:** Spearman’s rank correlations between biomarker concentration differences and doxorubicin dose intensity measures

	Doxo *C*_max_ (of corresponding sampling period)	Doxo AUC (of corresponding sampling period)	Doxo AUC (sum of all doxo administrations from first to second sampling period)	Doxo dose/BSA (sum of all doxo administrations from first to second sampling period)
NT-proANP (sample B)–NT-proANP (sample A)	−0.276 (*p* = 0.009)	−0.037 (*p* = 0.722)		
NT-proANP (sample D)–NT-proANP (sample C)	−0.207 (*p* = 0.052)	−0.310 (*p* = 0.767)		
BNP (sample B)–BNP (sample A)	−0.382 (*p* < 0.001)	−0.204 (*p* = 0.057)		
BNP (sample D)–BNP (sample C)	−0.306 (*p* = 0.004)	−0.066 (*p* = 0.536)		
NT-proBNP (sample B)–NT-proBNP (sample A)	−0.219 (*p* = 0.041)	−0.079 (*p* = 0.454)		
NT-proBNP (sample D)–NT-proBNP (sample C)	−0.214 (*p* = 0.046)	0.079 (*p* = 0.452)		
cTnT (sample E)–cTnT (sample A)			0.104 (*p* = 0.348)	0.181 (*p* = 0.1)
cTnI (sample E)–cTnI (sample A)			0.233 (*p* = 0.048)	0.262 (*p* = 0.025)

Furthermore, we could not detect any correlation between the difference in cardiac troponin plasma concentrations (between sampling times E and A) with the doxorubicin dose (normalized to BSA) administered as well as with the calculated sums of AUCs for that time frame (Table 2).

### Analysis of left ventricular shortening fraction (LVSF)

Data on the cardiac function were determined at the beginning (echo 1.1) and end (echo 2.2) of the trial. The mean LVSF values at echo 1.1 were 39 ± 8.1 % and the ones at Echo 2.2 37.6 ± 6.6 %, showing that for the doxo-trial patient collective subclinical cardiac function did not decrease severely throughout the trial period. Anyhow, six patients had throughout the study one LVSF value below 28 % and 20 patients had one or more LVSF values ≤30 %. Both are considered abnormal values. Furthermore, LVSF values of echo 2.2 negatively correlated with administered cumulative doxorubicin dose normalized to BSA [Spearman’s correlation: −0.567, *p* value <0.001].

## Discussion

It may be surprising to investigate a drug with decades of clinical experience, but doxorubicin highlights the complexity of pediatric drug development and the difficulties to implement this development into the drug regulatory framework. The drug is highly effective and its use has contributed significantly to increasing the cure rate for children with cancer. This success has mainly been driven by investigator-initiated trials in which treatment strategies and dosing rules were developed empirically rather than establishing dosing schemes based on systematic investigations of the influence of patient size and age.

Increasing cure rates were accompanied by increasing recognition of dose-dependent severe side effects, long-term toxicity and risk of chronic cardiac failure. Very young patients remained the group with the weakest evidence for age-appropriate dosing, although they are also the group with the highest uncertainties about the risks of doxorubicin exposure.

Acknowledging the need to study pharmacokinetics of doxorubicin in children, the EPOC consortium obtained funding within the 7th EU framework program. The resulting study was an add-on trial to the present disease-related national or European treatment protocols. These protocols vary largely concerning doxorubicin dosage, infusion times, chemotherapy regimen and concomitant medications, not only between the various cancer types but also between different countries. The doxo-trial thus comprised the wide range of existing clinical practice. To deal with this, heterogeneity was only possible by a) using a population PK approach, b) assuming a linear pharmacokinetic for doxorubicin and c) defining standard procedures for doxorubicin administration and blood sampling to minimize pre-analytical mistakes [16, 17]. With this approach, it was possible to combine data from patients receiving different doses and infusion times in one analysis and the primary question of the trial, whether there is a difference in the distribution of the clearances of the age groups <3 years and those 3 to <18 years could be addressed. A significant difference was identified with the BSA-normalized clearance being lower in the younger age group, meaning that similar dose calculation rules lead to higher dose intensity in the younger patients. As mentioned before, in most cancer therapy protocols, the dose is already reduced in the very young children, but dose reduction strategies vary largely between the different national or European treatment protocols. Our results strongly support these dose reductions. Anyhow, the analysis of PK parameters is only one of several aspects defining the optimal dosage. The actual clinical significance needs to be defined in specific clinical trials focusing on this issue. Our PK model can be used to develop a suggestion for an adequate and standardized dose reduction strategy. This is methodologically very demanding and needs scientific discussion taking pharmacological surrogates and clinical goals into account [18], which is ongoing right now. The suggested dose reduction strategy needs to be validated in clinical studies which is why it is an urgent need to bring these studies back on the agenda’s of the concerned trial groups.

Besides PK, the “doxo”-study explored the role of natriuretic peptides and cardiac troponins as potential early predictors for subclinical and eventually long-term cardiotoxicity. Although we observed a direct response for all three natriuretic peptides after drug application, we cannot exclude that this acute increase in plasma level may be due to stress from hospitalization and chemotherapy, raised fluid volume or other confounders at this point. Moreover, none or only weak correlations between raised biomarker levels and individual doxorubicin dose intensity (represented by AUC and *C*_max_) could be identified. Cardiac troponin did not directly respond to single doxorubicin applications, but plasma concentrations determined 1–2 weeks after doxorubicin administration showed a slight but statistically noticeable increase. Furthermore, cardiac troponin concentrations correlated with administered cumulative doxorubicin dose, which is the major risk factor for doxorubicin-induced cardiotoxicity. These observations match to the report from Lipshultz et al. observing an increase in the percentage of patients with at least one elevated cTnT level with time of cancer treatment [19] and might be a hint that cTnT might be a suitable prognostic marker for anthracycline-induced cardiotoxicity. It therefore would be interesting to introduce measurements of cTnT or cTnI, which largely correlated in our study, into further studies, e.g., disease-specific cancer trials, and follow changes in blood concentrations over the whole chemotherapy for each patient, since a big limitation of our study with respect to the pharmacodynamic surrogates was the short trial duration for each patient. Another limitation was the small number of patients. Since echocardiography itself is not a very sensitive prognostic marker for late cardiac effects, verification of a cardiotoxic biomarker can only be done by correlation to late cardiac events. Since these occur in less than 10 % of patients, the number of patients recruited in the doxo-trial is too low to allow reliable predictions. EPOC is nevertheless discussing possibilities with late effect groups and authorities on how long-term follow-up can be organized. It would be interesting to recheck the amount of doxorubicin dose intensity (AUC and Cmax) as well as biomarker blood levels for patients with late subclinical or clinical cardiac events.

In summary, the trial substantiated the existing dose reduction strategies for infants and should serve as a step forward toward standardized age-dependent dosing for young children. In addition, dose calculation, age-dependent pharmacodynamics and clinical needs require an intensified focus and prospective evaluation within the respective clinical trials.

## Electronic supplementary material

Below is the link to the electronic supplementary material.

Supplementary material 1 (PDF 102 kb)

Supplementary material 2 (PDF 131 kb)

Supplementary material 3 (PDF 204 kb)

Supplementary material 4 (PDF 247 kb)
