# Supplementary material for: Pharmacokinetic and pharmacodynamic study of doxorubicin in children with cancer: results of a “European Pediatric Oncology Off-patents Medicines Consortium” trial
Source: Cancer Chemother Pharmacol. 2016 Oct 21;78(6):1175–84. doi: 10.1007/s00280-016-3174-8 (PMC5114325; doi:10.1007/s00280-016-3174-8)
Supplement: Supplementary file 1 — Supplementary material 1 (PDF 102 kb) [file 280_2016_3174_MOESM1_ESM.pdf]

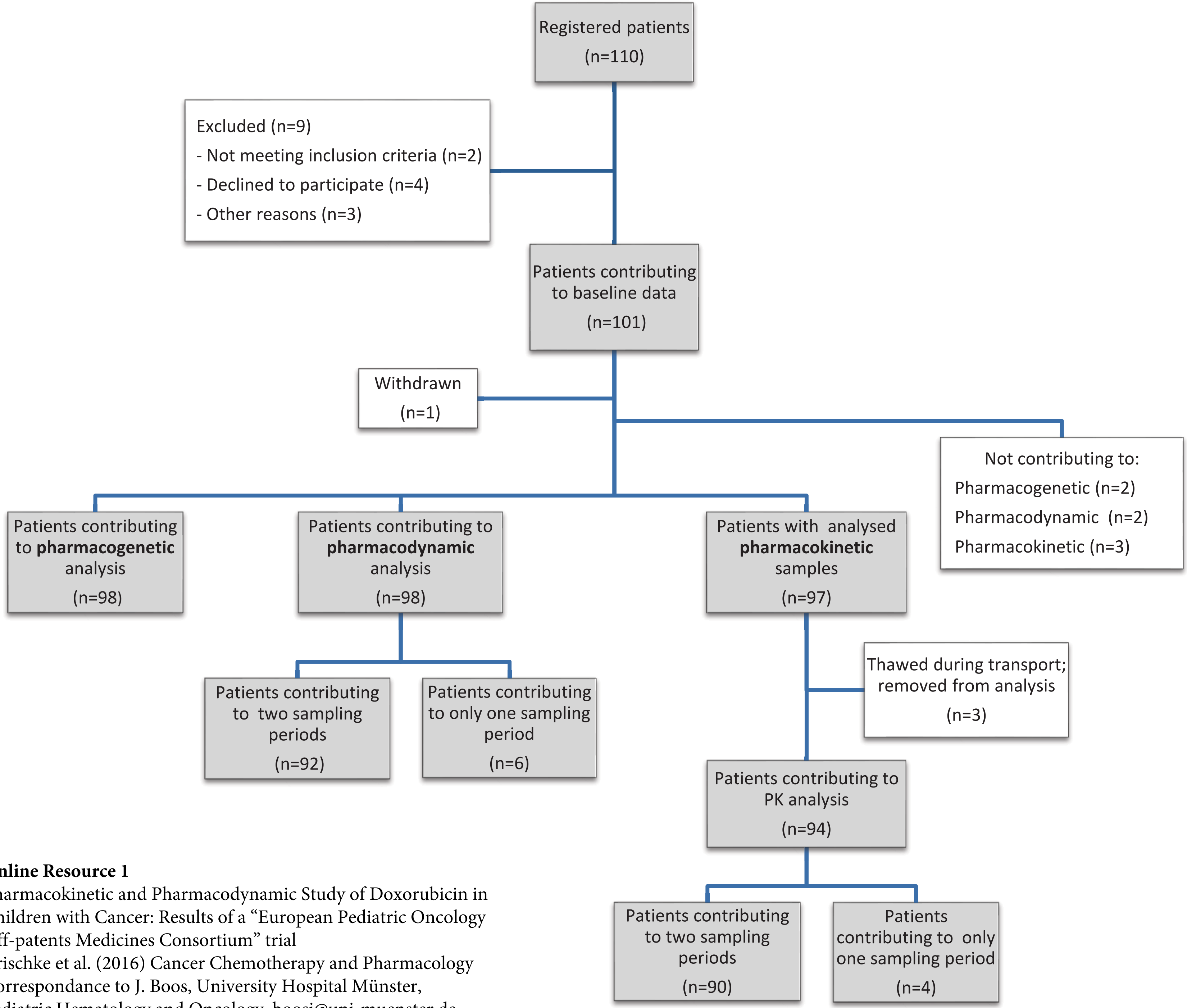

Online Resource 1

Pharmacokinetic and Pharmacodynamic Study of Doxorubicin in Children with Cancer: Results of a “European Pediatric Oncology Off-patents Medicines Consortium” trial

Krischke et al. (2016) Cancer Chemotherapy and Pharmacology

Correspondance to J. Boos, University Hospital Münster, Pediatric Hematology and Oncology, boosj@uni-muenster.de
