# Supplementary material for: Pharmacokinetic and pharmacodynamic study of doxorubicin in children with cancer: results of a “European Pediatric Oncology Off-patents Medicines Consortium” trial
Source: Cancer Chemother Pharmacol. 2016 Oct 21;78(6):1175–84. doi: 10.1007/s00280-016-3174-8 (PMC5114325; doi:10.1007/s00280-016-3174-8)
Supplement: Supplementary file 2 — Supplementary material 2 (PDF 131 kb) [file 280_2016_3174_MOESM2_ESM.pdf]

Online Resource 2:

Doxorubicin dosages and infusion times used in various paediatric oncology treatment protocols in Germany (GER), France (F), Italy (I) and United Kingdom (UK)

Tumour type	Country	Protocol	Protocol block	Doxorubicin dose	Infusion time
**Wilms tumour**	GER, UK, F, I	SIOP WT 2001	AVD: Stage II, III, IV	50mg/m²	≥ 6h (GER)<br>4-6h (UK, I, F)
	GER, UK, F, I	SIOP WT 2001	High Risk: Stage II, III, IV	50mg/m²	≥ 6h (GER)<br>4-6h (UK, I, F)
**Neuroblastoma**	GER	NB 2004	a) N4	a) 15mg/m²	a) 30 min
			b) N6	b) 30mg/m²	b) 4 h
	UK, I, F	HR-NBL-1 (SIOPEN)	TVD (high risk non responders)	22.5mg/m^2/day x2 (50mg/m^2 total)	48 h
	F	SFCE - (NB02 “like”) Non resectable localized NB	CADO	1mg/kg x2	6h
**Ewing sarcoma**	GER, UK, F	EURO EWING 99	VIDE	20mg/m²	4 h
	I	ISG/AIEOP EW-1	a) VAC<br>b) VAI	40 mg/m^2	4h

Correspondance to J. Boos, University Hospital Münster, Pediatric Hematology and Oncology, boosj@uni-muenster.de

Tumour type	Country	Protocol	Protocol block	Doxorubicin dose	Infusion time
**RMS /soft tissue sarcoma**	GER	CWS 2002 P (soft-tissue sarcoma, incl. rhabdo-myosarcoma)	VAIA III	2x 20mg/m²	2x 3 h
	UK, F, I	RMS 2005 (STS 2006 04) rhabdo-myosarkoma	High risk	30mg/m² x2 (=60mg/ m²/course)	4h
	UK, F, I	NRSTS 2005 (STS 2006 03) Localised, Non-Rhabdo Soft tissue sarcomas	IA	37.5mg/m²/dayx2 (=75mg/m²/course)	4-6h (UK)<br>10mg/hour (F)<br>1-4h (I)
**ALL**	GER	ALL-BFM 2000	protocol II	30mg/m² (weekly, 4 doses)	1 h
	I	AIEOP LLA 2000	protocol II	30mg/m² (weekly, 4 doses)	1 h
	UK	UKALL2003	Delayed Intensification	25mg/ m²	1 h
	F	FRALLE 2000	a) Consolidation	40mg/m²	1 h
			b) Intensification		1 h

Pharmacokinetic and Pharmacodynamic Study of Doxorubicin in Children with Cancer: Results of a “European Pediatric Oncology Off-patents Medicines Consortium” trial
Krischke et al. (2016) Cancer Chemotherapy and Pharmacology
Correspondance to J. Boos, University Hospital Münster, Pediatric Hematology and Oncology, boosj@uni-muenster.de
