# Supplementary material for: Pharmacokinetic and pharmacodynamic study of doxorubicin in children with cancer: results of a “European Pediatric Oncology Off-patents Medicines Consortium” trial
Source: Cancer Chemother Pharmacol. 2016 Oct 21;78(6):1175–84. doi: 10.1007/s00280-016-3174-8 (PMC5114325; doi:10.1007/s00280-016-3174-8)
Supplement: Supplementary file 3 — Supplementary material 3 (PDF 204 kb) [file 280_2016_3174_MOESM3_ESM.pdf]

Online Resource 3

Detected SNPs in Genes involved in Doxorubicin metabolism and transport

Gene	Polymorphism	Wild Type	Hetero-zygous	Homo-zygous	missing
**NQO1**	**C609T - rs1800566**	58	35	4	1
	**rs1143684**	57	33	7	1
	**insertion/deletion**	78	14	0	6
**ABCB1**	**rs1128503**	32	46	18	2
	**rs2032582**	31	46	17	4
	**rs1045642**	27	43	26	2
**SLC22A16**	**rs723685**	73	22	1	2
	**rs12210538**	53	35	8	2
	**rs714368**	55	36	5	2
	**rs6907567**	55	36	5	2
**CBR1**	**rs9024**	81	16	1	0
**CBR3**	**rs8133052**	18	58	19	3
	**rs1056892**	44	44	10	0
**ABCC1**	**rs4148350**	87	10	0	1
	**rs246221**	43	44	10	1
**SLC28A3**	**rs7853758**	72	22	2	2
**UGT2B7**	**hCV32449742-C_32449742_20**	19	42	35	2

Correspondance to J. Boos, University Hospital Münster, Pediatric Hematology and Oncology, boosj@uni-muenster.de
