# Supplementary material for: Pharmacokinetic and pharmacodynamic study of doxorubicin in children with cancer: results of a “European Pediatric Oncology Off-patents Medicines Consortium” trial
Source: Cancer Chemother Pharmacol. 2016 Oct 21;78(6):1175–84. doi: 10.1007/s00280-016-3174-8 (PMC5114325; doi:10.1007/s00280-016-3174-8)
Supplement: Supplementary file 4 — Supplementary material 4 (PDF 247 kb) [file 280_2016_3174_MOESM4_ESM.pdf]

Online Resources 4

Spearman's rank correlations between different cardiotoxicity biomarkers.

	**Troponin T**	**Troponin I**	**NT-proANP**	**BNP**
Sampling Time A				
**Troponin I**	**0.713**			
**NT-proANP**	0.454	0.282		
**BNP**	0.216	0.206	**0.567**	
**NT-proBNP**	0.455	0.406	**0.514**	**0.604**
Sampling Time B				
**Troponin I**	**0.589**			
**NT-proANP**	0.413	0.293		
**BNP**	0.310	0.285	**0.682**	
**NT-proBNP**	0.395	0.273	**0.631**	**0.746**
Sampling Time C				
**Troponin I**	**0.654**			
**NT-proANP**	0.473	0.391		
**BNP**	0.200	0.195	**0.485**	
**NT-proBNP**	0.332	0.428	**0.538**	**0.623**
Sampling Time D				
**Troponin I**	**0.576**			
**NT-proANP**	0.438	0.315		
**BNP**	0.342	0.281	**0.753**	
**NT-proBNP**	0.436	0.446	**0.673**	**0.811**
Sampling Time E				
**Troponin I**	**0.688**			
**NT-proANP**	0.481	0.225		
**BNP**	0.225	0.290	**0.525**	
**NT-proBNP**	0.387	0.331	**0.592**	**0.757**

Pharmacokinetic and Pharmacodynamic Study of Doxorubicin in Children with Cancer: Results of a "European Pediatric Oncology Off-patents Medicines Consortium" trial
Krischke et al. (2016) Cancer Chemotherapy and Pharmacology
Correspondance to J. Boos, University Hospital Münster, Pediatric Hematology and Oncology, boosj@uni-muenster.de
